# Ischemic stroke complicating thrombolytic therapy with tenecteplase for ST elevation myocardial infarction: two case reports

**DOI:** 10.1186/s13256-017-1322-3

**Published:** 2017-06-11

**Authors:** Salim Arous, Meryem Haboub, Mohamed El Ghali Benouna, Tarik Bentaoune, Rachida Habbal

**Affiliations:** Department of Cardiology, Ibn Rushd University Hospital, Casablanca, Morocco

**Keywords:** Ischemic stroke, Thrombolysis, Myocardial infarction

## Abstract

**Background:**

Hemorrhagic complications are quite common in the rare cases where thrombolysis is performed. Ischemic stroke in the aftermath of thrombolysis for a ST elevation myocardial infarction is a very rare and paradoxical complication. With these observations in mind we report two interesting cases of ischemic stroke which occurred after fibrinolytic therapy with tenecteplase for a ST elevation myocardial infarction.

**Case presentation:**

The first case was a 56-year-old African man who presented with an acute infero-basal ST elevation myocardial infarction 6 hours after chest pain onset. Thrombolysis with tenecteplase was performed and few minutes later an ischemic stroke occurred. The second patient was a 65-year-old African man who presented with an acute infero-basal ST elevation myocardial infarction 5 hours after chest pain onset. Thrombolysis was performed and 10 hours later an ischemic stroke occurred.

**Conclusions:**

Hemorrhagic stroke is not the only complication of thrombolysis, ischemic stroke can occur even if it is an extremely rare complication. The two cases on which we report shed light on the association between fibrinolytic therapy and ischemic stroke, the pathophysiology of which is not well understood.

## Background

ST elevation myocardial infarction (STEMI) is due to complete occlusion of the coronary artery. The gold standard therapy for STEMI is recanalization of the infarct-related coronary artery as soon as possible by pharmacological or mechanical means. The indication for this is STEMI presenting within 12 hours of symptom onset.

Primary percutaneous coronary intervention (PCI) is superior to thrombolytic therapy (TT), but there are many limitations to PCI such as the absence of a nearby angioplasty center. TT is therefore an effective treatment of choice in STEMI, as it is easy to perform anywhere and at any time [1].

The efficacy and safety of various thrombolytic agents have been well documented in large clinical trials. The most feared complication of TT is intracranial hemorrhage which is well documented in the literature. Ischemic stroke in the aftermath of TT for STEMI worsens the patient’s prognosis; it is a paradoxical, very rare complication, the pathophysiology of which is not well understood [2]. Hence, we report two interesting cases of ischemic stroke after TT for STEMI to shed light on the association between TT and ischemic stroke.

## Case presentation

### Case report 1

**Fig. 1:**
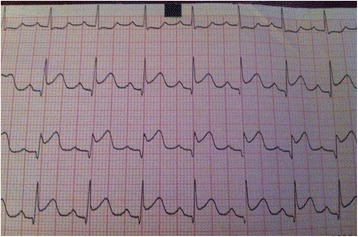
Electrocardiogram showing ST-segment elevation in inferior leads

A 56-year-old African man presented to our emergency department 6 hours after severe chest pain onset. He was a tobacco smoker, diabetes status unknown, neither hypertensive nor dyslipidemic, and had no history of stroke. He had no personal or family medical history, including heart disease or heart rhythm disorder, and was not under any treatment prior to diagnosis. He was from a low socio-economic level. An electrocardiogram (EKG) showed a sinus rhythm with ST-segment elevation in inferior and posterior leads (Fig. 1).

At admission he was conscious, Glasgow Coma Scale (GCS) of 15/15, without motor deficit or sensory disorder; his chest pain was constrictive irradiating to his two upper limbs without syncope. Blood pressure (BP) on admission was 140/85 mmHg symmetrical, regular rhythm at 60 beats per minute (bpm), heart sounds clearly auscultated with no heart or carotid murmur, there was no murmur of mitral insufficiency or ventricular septal defect, no signs of heart failure including no crackles, and he was without edema of his lower limbs or turgor of his jugular veins leading to a right ventricular infarction. His peripheral pulses were perceived symmetrically. The rest of the examination was strictly normal. Acute inferior and posterior STEMI was diagnosed and intravenously administered TT using tenecteplase (intravenous bolus of 30 mg considering a weight of 54 kg) was performed with adjuvant antithrombotic medication (aspirin, clopidogrel, and enoxaparin).

**Fig. 2:**
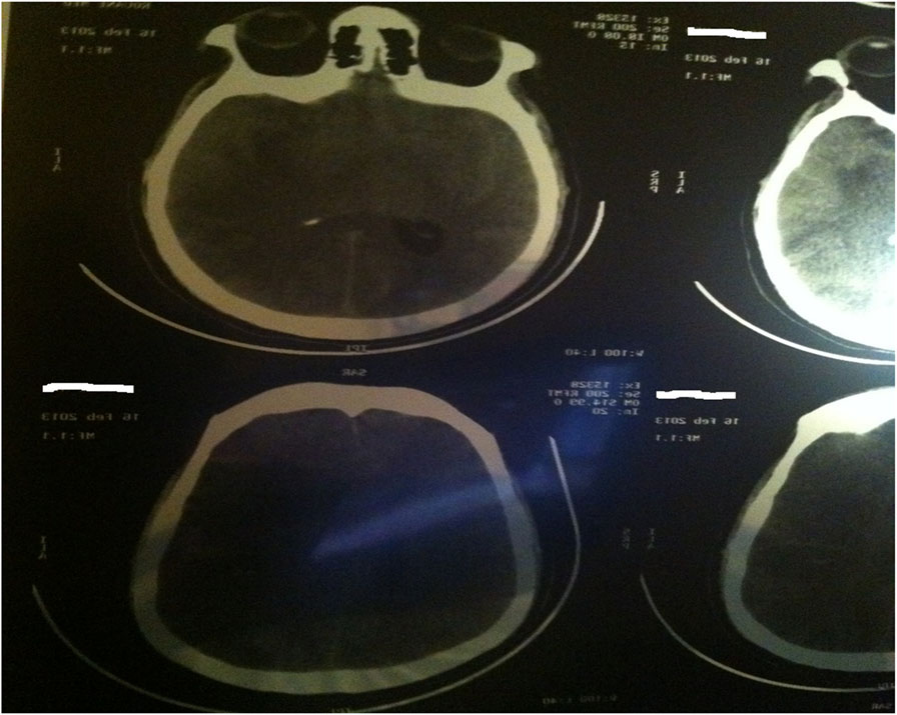
Cerebral computed tomography showing frontal, temporal, and parietal left ischemic stroke

A few minutes later, he developed motor aphasia and right hemiplegia with altered level of consciousness: GCS of 12/15. The first cerebral computed tomography (CT) performed 1 hour later was normal and the second one performed 12 hours later showed a frontal, temporal, and parietal left ischemic stroke with a hemorrhagic infarct (Fig. 2). On an EKG, the ST-segment elevation regressed more than 50% at 60 minutes after TT. An echocardiographic examination (Vivid 6S) was performed following the therapy. It revealed a left ventricle ejection fraction (LVEF) at 50%, no mitral insufficiency, pulmonary artery pressure at 36 mmHg, no thrombus was detected in any cavity, no ventricular septal defect, and no pericardial effusion. An echo-Doppler of the supra-aortic trunks was not performed. The laboratory findings were as follows: troponin Ic, 18 ng/ml; normal liver function tests; glomerular filtration rate (GFR) by Modification of Diet in Renal Disease (MDRD) method at 85 ml/1.73 m^2^ body surface area (BSA) per minute; hemoglobin, 13.5 g/dl; platelets count, 500,000/mm^3^; plasmatic fibrinogen level, 2.49 g/l before TT and 5.89 g/l 12 hours after; white blood cells count (WBC), 15,000 mm^3^; and C-reactive protein (CRP), 72 mg/l. Antithrombotic medication was discontinued. His neurological condition worsened progressively and, after 1 week, he died.

### Case report 2

**Fig. 3:**
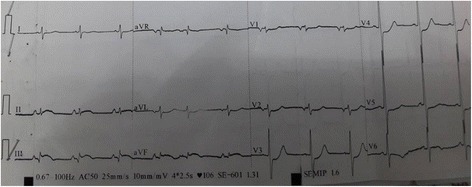
Electrocardiogram showing ST-segment elevation in inferior leads and a mirror aspect in V1 to V4

A 65-year-old African man presented to our emergency department 5 hours after severe chest pain onset. He is a tobacco smoker, diabetes status unknown, neither hypertensive nor dyslipidemic, and had no history of stroke. He had no personal or family medical history, including heart disease or heart rhythm disorder, and he was not under any treatment prior to diagnosis. He was from a low socio-economic level. An EKG showed a sinus rhythm with ST-segment elevation in inferior and posterior leads (Fig. 3). At admission he was conscious, GCS of 15/15, without motor deficit or sensory disorder; his chest pain was constrictive irradiating to his two upper limbs without syncope. BP on admission was at 190/120 mmHg symmetrical, pulse rate at 122 bpm, heart sounds clearly auscultated, no heart or carotid murmur, there was no murmur of mitral insufficiency or ventricular septal defect, no signs of heart failure including no crackles, and he was without edema of his lower limbs or turgor of his jugular veins leading to a right ventricular infarction. His peripheral pulses were perceived symmetrically. The rest of the examination was strictly normal. Acute inferior and posterior STEMI was diagnosed and intravenously administered TT using tenecteplase (intravenous bolus of 35 mg considering a weight of 65 kg) was performed after lowering BP at 150/90 mmHg, associated with aspirin 300 mg administered orally and clopidogrel 300 mg administered orally. It is important to note that the first dose of heparin was missed. Echocardiographic examination (Vivid 6S) was performed following the therapy. It revealed segmental wall motion abnormalities, LVEF at 50%, no mitral valve insufficiency, pulmonary artery pressure at 29 mmHg, and no thrombus or any mechanical complication was detected. One hour after TT, his chest pain and more than 50% of ST-segment elevation resolved with no hemorrhagic complications. The laboratory findings were as follows: troponin Ic, 9 ng/ml; normal liver function tests; serum creatinine level, 14.9 mg/l; estimating a GFR at 50.5 ml/1.73 m^2^ BSA per minute using MDRD; hemoglobin, 16.3 g/dL; hematocrit, 49.5%; platelets count, 281,000/mm^3^; plasmatic fibrinogen level, 7.4 g/l; WBC, 14,900/mm^3^; and CRP, 62 mg/l.

**Fig. 4:**
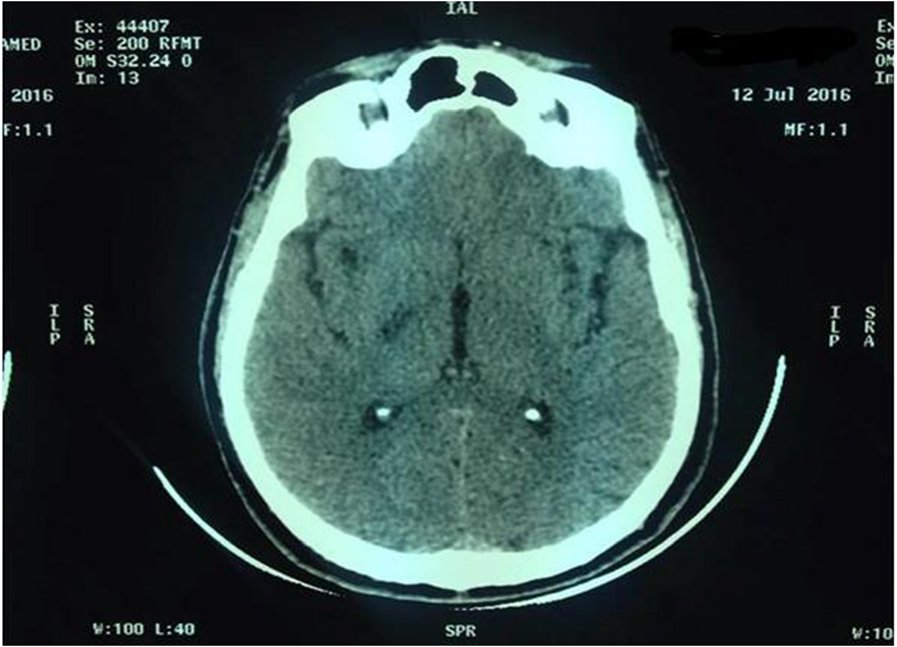
Cerebral computed tomography showing a right internal capsule ischemic stroke

Ten hours later, he developed a left hemiparesis with motor aphasia without sensory disturbance and drowsiness: GCS of 14/15. The first cerebral CT performed 3 hours later was normal and the one performed 24 hours later showed right internal capsule ischemic stroke without hemorrhagic infarct (Fig. 4). An echo-Doppler of the supra-aortic trunks was normal. The case was discussed with neurologists and aspirin 75 mg administered orally once daily, clopidogrel 75 mg administered orally once daily, and unfractionated heparin, which was monitored by partial thromboplastin time (PTT), were continued. His neurological condition improved (level of consciousness and motor deficit). He was discharged after 12 days of hospitalization; physiotherapy and neurological follow-up were planned. One month after discharge, PCI using a bare-metal stent (BMS) was performed on a circumflex coronary artery lesion.

He was followed-up regularly after discharge every 2 months. At 1 year of follow-up, clinically he no longer presents a deficit, he is without recurrence of chest pain, and the ischemic image at a cerebral control scan disappeared. No hemorrhagic complications were developed under dual antiplatelet therapies (DAPT). Clopidogrel was stopped after 6 months.

## Discussion

STEMI, caused by acute occlusion of the infarct-related coronary artery, is an emergency condition. The primary therapy is restoration of full antegrade flow by either PCI or TT in patients presenting within 12 hours of symptom onset. Although primary PCI is superior to TT in patients with STEMI, there are many limitations in clinical practice. TT is therefore an effective treatment of choice in STEMI as it is easy to perform anywhere and at any time.

All thrombolytic agents share a common mechanism of activating plasminogen into plasmin, which in turn activates the fibrin degradation pathway. The efficacy and safety of various thrombolytic agents have been well documented in large clinical trials [2]. TT decreases mortality in patients with STEMI, but as experience with thrombolytic agents grows, the potential risk of serious side effects becomes more apparent. The most feared complication is bleeding (global incidence of bleeding is 10%), especially intracranial hemorrhage which occurs in 0.9% of patients treated with tissue plasminogen activator (tPA), and it is well documented [3, 4].

Bleeding after fibrinolytic treatment is due to the depletion of clotting factors and lysis of recently formed hemostatic plugs [5]. However, embolic cerebral infarction after TT is not well documented in the literature, which is the reason why we have reported our cases.

Before the era of TT, the incidence of stroke during a hospital stay after acute coronary syndrome (ACS) ranged from 0.7 to 2.2% with a mortality rate at 46%. Hachet and colleagues reported that typical cases occurred during the first week and 33% during the first 24 hours [6]. These rates can be confirmed by the observational studies of American and Swedish patient registries which show the results of representative patient populations. The evaluation of the American Nationwide Inpatient Sample revealed a rate of neurologic complications of 2% (ischemic stroke 1.5%, transient ischemic attack 0.3%, and hemorrhagic stroke 0.2%) [7]. Predictive factors of stroke after acute myocardial infarction (AMI) were: anterior or apical wall motion abnormalities, atrial arrhythmias, cardiogenic shock, and history of stroke. Most cases were attributed to cerebral embolization [8].

Ischemic stroke after TT for STEMI is paradoxical and rare. A multivariate analysis showed a significantly low risk after TT performed within 15 minutes and a low but not significant risk after primary PCI performed within 90 minutes of first medical contact. Despite the use of fibrinolytics and primary PCI, the incidence of left ventricular thrombus formation seems to be lower but remains substantial [9].

The Trial of ORG 10172 in Acute Stroke Treatment (TOAST trial) studied ischemic strokes during hospital stay at the acute phase of myocardial infarction: 60% was of cardioembolic origin and 36% were of undetermined pathogenesis [6].

The underlying pathophysiological processes of ischemic strokes after AMI are multifactorial. Ischemia itself induces a systemic procoagulant effect, facilitating cardiac thrombus formation and embolization. Furthermore, ischemia results in the release of inflammatory cytokines; this might trigger the destabilization and rupture of plaques in the cerebral circulation. Therefore, complex and unstable carotid plaques are common in patients with AMI (42% versus 8% in patients with stable angina) [10]. These processes explain the beneficial effect of a fast restoration of coronary flow with respect to the risk of ischemic strokes following AMI [11].

Cardioembolic strokes after AMI are mainly caused by atrial fibrillation and left ventricular thrombi [9]. Cardiac embolic ischemic stroke after fibrinolytic treatment for ACS with ST-segment elevation is rare. Some cases are reported in the literature, explained by a paroxysmal atrial fibrillation or by thrombophilia by protein C deficiency and mutation C677T in methylenetetrahydrofolate reductase (MTHFR) [2, 7].

There is also a procoagulant state of thrombolytic treatment which can explain the occurrence of a stroke after thrombolysis, hence the interest in systematically giving heparin, unfortunately that was not done with our second patient.

Left ventricle thrombi formation is mainly caused by an akinetic segment of the left ventricular myocardium which can fragment and migrate to the brain after thrombolysis. It is a common complication in large anterior and apical myocardial infarction with a low LVEF (less than 40%) and the thrombotic risk increases with decrease in LVEF. The thrombi usually occur within 2 weeks after AMI; a median of 5 days after the acute event. In the prethrombolytic era, ischemic stroke after STEMI was a common complication and the incidence of thrombus formation was reported to range from 20 to 55% in patients with an anterior STEMI [9].

Crenshaw *et al*. showed a significant risk of ischemic stroke after AMI with atrial fibrillation treated with TT [11]. Another strong predictor of an increased stroke risk following AMI is the presence or new onset of atrial fibrillation. Data from the Framingham Study show that a myocardial infarction, or even ischemia, is a risk factor for new onset of atrial fibrillation [12]. Following stent implantation, patients with atrial fibrillation are at risk of both stroke and stent thrombosis and, therefore, have an indication for triple therapy [11].

In total, several mechanisms may explain the occurrence of an ischemic stroke in the course of a thrombolysis of a STEMI. In some cases it has been explained by the procoagulant effect of thrombolytics. Embolization of microthrombi formed at the left ventricle or from carotid plaques following thrombolysis has also been reported. Some cases were explained by a paroxysmal atrial fibrillation or by thrombophilia by protein C deficiency and mutation C677T in MTHFR.

In our two cases, patients were in sinus rhythm, the STEMI was inferior and posterior, LVEF was 40% or more, and no intracavitary thrombus was detected. The ischemic stroke could be explained by inflammatory reaction due to ischemia causing left ventricle microthrombi and it is presumed that TT induced lysis, fragmentation, and embolization of microthrombi in the cerebral circulation. Inflammatory reaction due to ischemia could also explain destabilization of carotid plaques. In addition, our second patient missed the first dose of heparin.

## Conclusions

Hemorrhagic stroke is not the only complication of thrombolysis, ischemic stroke can occur even if it is an extremely rare complication. Those cases represent an extremely rare clinical condition on which we report to shed light on the association between fibrinolytic therapy and ischemic stroke.

Patients receiving TT for the treatment of STEMI should receive immediate antithrombotic co-therapies, have constant neurological re-evaluation, and clinicians must be prepared to handle such complications in a timely manner.

Additional studies are needed to clarify the benefice of thrombolysis in high risk patients, especially the elderly or ones with a history of stroke.
